# Should a single blastocyst transfer policy be a clinical decision or should it depend on the embryological evaluation on day 3?

**DOI:** 10.1186/1477-7827-9-60

**Published:** 2011-05-05

**Authors:** Dominic Stoop, Lisbet Van Landuyt, Etienne Van den Abbeel, Michel Camus, Greta Verheyen, Paul Devroey

**Affiliations:** 1 Centre for Reproductive Medicine, University Hospital, Vrije Universiteit Brussel (Free University of Brussels), Laarbeeklaan 101, 1090 Brussels, Belgium

## Abstract

**Background:**

Single blastocyst transfer has the advantage of maximizing the fresh single pregnancy rate. However, in patients with a low number of good quality embryos on day 3, it remains unclear whether immediate embryo transfer or further embryo culture with blastocyst transfer is the most preferable option.

**Methods:**

A retrospective cohort study was carried out in which the outcome of 590 fresh in vitro fertilization (IVF) cycles over a 15 months period and their cryo cycles were analyzed. A total of 341 patients cycles had an elective day 5 strategy independent of intermediate embryo evaluation while another 249 patients underwent a day 5 embryo transfer only if at least four embryos were available on day 3. Blastocyst vitrification was performed using a closed high security system.

**Results:**

Demographics, stimulation parameters and embryological data were comparable in the two groups. Patients in the elective day 5 group had a lower fresh transfer rate (90.62% vs. 95.18%, p < 0.05) as compared to patients with a day 3 or day 5 embryo transfer policy. No difference was observed in the fresh live birth rate and multiple pregnancy rate per initiated cycle (32.84% vs. 28.92%; 1.17% vs 0%) The projected cumulative ongoing pregnancy rate compensating for double counting in case subjects have more than one pregnancy is not different (42.58% vs. 39.84%).

**Conclusions:**

Despite lower fresh transfer rates, elective single blastocyst transfer yields a similar projected cumulative ongoing pregnancy rate as in a policy with cleavage stage or blastocyst transfer depending on a good quality embryo count on day 3.

## Background

Single embryo transfer (SET) has been advocated as a strategy to reduce the frequency of multiple births after in vitro fertilization [1]. It has been established that the significantly lower pregnancy rate achieved after single cleavage stage embryo transfer as compared to a double embryo transfer can be compensated by pregnancies resulting from the first thawed cycle [2]. This finding underscores the important role of cryopreservation in enhancing the total reproductive potential of a single cycle [3]. The introduction of the vitrification technique as an efficient cryopreservation technique promises to further increase the impact of cryo-technology on the cycle specific pregnancy rates.

Fresh pregnancy rates can be optimized by performing blastocyst transfer as such approach yields higher live birth rates than single cleavage stage embryo transfer [4,5]. However, a systematic blastocyst transfer policy holds the risk of cancelled embryo transfers, as extended embryo culture may be unsuccessful. Morphological scoring of embryo quality on day 3 may therefore offer a tool to identify patients that would potentially benefit from further embryo culture [6]. The threshold of four good embryos on the third day appeared to be a reassuring indication that the patient will undergo an embryo transfer [6].

The aim of present study is to compare two approaches, both aimed at a single blastocyst transfer. The outcome after elective blastocyst transfer was compared with the outcome of a blastocyst transfer or a cleavage stage transfer depending on whether the criteria of four good embryos on the third day were met. Moreover, the additional cryo-pregnancies and projected cryo-pregnancies through blastocyst vitrification have been evaluated.

There is an ongoing debate on the microbiological safety of cryopreservation since the report of a hepatitis B transmission from contaminated cryopreserved bone marrow and reports on potential contamination of reproductive cells in experimental conditions [7,8]. Although no clinical reports of contamination have been reported for reproductive cells and tissues, research is ongoing aimed at eliminating such risks. In this study we evaluate the effectiveness of blastocyst vitrification performed with a high security vitrification system preventing all contamination risk during vitrification and storage.

## Methods

### Study design

A retrospective cohort study was performed. All fresh IVF or ICSI treatment cycles were performed in a 15 months period from March 2008 until June 2009. All cycles where the physicians' initial treatment plan envisaged eSET with either a fixed day 5 transfer or with a day 3 or day 5 transfer depending on the embryo quality on day 3 were included. Only the first treatment cycle was considered in case patients were undergoing multiple attempts.

### Embryo culture, evaluation and selection day of transfer

Sperm preparation, IVF/ICSI procedures and embryo culture were carried out as described by Van Landuyt et al. [9]. Oocytes and embryos were cultured in sequential media and cultured at 37°C in an atmosphere of 6% CO_2_, 5% O_2 _and 89% N_2_. Fertilization was assessed 16-19 h after insemination or injection. On the morning of day 3 embryos were transferred from cleavage medium to blastocyst medium. Embryo quality was assessed daily was scored as described by Papanikolaou et al., [6] according to the following parameters: number of blastomeres, rate of fragmentation, multinucleation of the blastomeres and early compaction. Blastocyst quality on day 5/6 was assessed according to the criteria of Gardner and Schoolcraft [10].

### Embryo cryopreservation procedures

For patients receiving transfer on day 3, supernumerary cleavage stage embryos were cryopreserved and thawed using standard slow freezing as described by Van den Abbeel et al., (2005). Blastocyst vitrification was performed using closed CBS-VIT High Security (HS) straws (Cryo Bio system) in combination with DMSO-EG-S as the cryoprotectants (Irvine Scientific^R ^Freeze Kit). The vitrification procedure was carried out at room temperature (between 22 and 27°C). Blastocysts were vitrified one by one. The blastocyst was first incubated for 2 min in a 50 μl droplet of HEPES-buffered culture medium. Then, the blastocyst was brought in a 50 μl droplet of equilibration solution (ES) containing 7.5% (v/v) DMSO and 7.5% (v/v) ethylene glycol and incubated for 10 min. The blastocyst was then transferred consecutively into four 25-μl droplets with vitrification solution (VS) containing 15% (v/v) DMSO and 15% (v/v) ethylene glycol. The blastocyst was incubated for 5 sec in droplet 1 and 2 and for 10 sec in the 3^rd ^droplet. The blastocyst was then transferred to the fourth droplet and immediately loaded onto the CBS-HS straw. The straw was heat sealed and plunged into liquid nitrogen. The total time needed to vitrify the blastocyst starting from VS droplet 1 to the loading of the straw and plunging into liquid nitrogen did not exceed 90 sec.

On the day of transfer blastocysts were warmed one by one until one blastocyst was available for transfer. Blastocysts were warmed randomly, independent of the blastocyst stage or ICM/TE quality prior to vitrification. For warming, the Irvine Scientific^R ^Thaw Kit was used. A Petri-dish containing two 25 μl droplets with thawing solution (TS, 1 M sucrose in Hepes buffered HTF medium supplemented with 20% DSS) was kept at 37°C. For warming the straw, the straw was transferred from the LN_2 _storage container to a transport dewar filled with liquid nitrogen. After cutting the straw and pulling the capillary from the straw, the gutter was placed in the first droplet with TS and the blastocyst was released from the gutter and kept at room temperature. The blastocyst was incubated for two times 1 min at room temperature in the two TS droplets. The blastocyst was then transferred to the first of two dilution solution droplets of 25 μl (DS; 0.5 M sucrose in Hepes buffered HTF medium supplemented with 20% DSS) and after that incubated for 2 min in a second DS droplet. Finally, the blastocyst was washed in 3 droplets (25 μl) of washing solution (Hepes buffered HTF medium supplemented with 20% DSS), each for 3 min. The blastocyst was transferred to a culture dish with blastocyst medium to assess its morphological survival. If the blastocyst was severely or completely damaged, a new one was warmed immediately. If the blastocyst was fully intact or showed moderate damage, expansion and re-expansion was assessed 1-2 hours later. If the morphological quality of the blastocyst was regressing or no signs of re-expansion were present, a second blastocyst was warmed until one blastocyst was suitable for transfer i.e. with at least moderate survival and expansion/re-expansion of the blastocyst

### Statistical analysis

All data management and statistical analysis were performed using the Statistical Package for the Social Sciences version 17.0 (SPSS Inc. Chicago, OL, USA). Student't t-test were performed on continuous variables to determine differences in mean scores and standard deviation (SD). Categorical variables were analyzed using Chi square analysis. A significant level of 0.05 was accepted throughout.

## Results

### Patient demographics and stimulation characteristics (Table 1)

**Table 1:** Patient demographics and stimulation characteristics

	eSET with elective day 5 transfer	eSET with transfer day 3 or 5		Statistical significance
No. subjects	341	249		-
Age (years)	29.94 (3.54)	31.20 (3.18)		NS
Rank of trial	0.29 (0.61)	0.39 (0.68)		NS
Days of stimulation	9.53 (2.09)	9.83 (2.22)		NS
Total gonadotropin dose (IU)	1745.95 (632.93)	1857.56 (685.55)		NS
		Day 3	Day 5	
No. subjects		135	102	
Age (years)		31.24 (3.23)	31.30 (3.11)	NS
Rank of trial		0.36 (0.66)	0.44 (0.73)	NS
Days of stimulation		9.94 (2.35)	9.78 (1.98)	
Total gonadotropin dose ((IU)		1907.56 (671.37)	1800.17 (704.74)	NS

Overall 590 treatment cycles have been performed during the observation period with 341 (58%) in the fixed day 5 group and 247 (42%) in the day 3 or day 5 group. The two groups were comparable with regard to age, rank of trial, duration of stimulation and the total dose of gonadotrophins administered.

### Fresh embryo transfer: embryology data (Table 2)

**Table 2:** Embryology data: fresh embryo transfer

	eSET with elective day 5 transfer	eSET with transfer day 3 or 5		Statistical significance
*Proportion IVF/ICSI as in vitro *fertilization procedure (%)				
IVF	44/341 (12.90)	24/247 (9.72)		NS
ICSI	297/341 (87.10)	223/247 (90.28)		NS
Mean No. cumulus-oocyte complexes (SD)	12.33 (7.23)	11.66 (7.35)		NS
Mean No. metaphase II oocytes	8.50 (6.26)	8.27 (6.46)		NS
Mean No. two pronuclei oocytes	7.46 (5.03)	7.11 (4.95)		NS
Embryos on day 3	2393	1690^1^		-
Embryos on day 5	1619	816^2^		
Embryos transferred fresh	309	237		-
Embryo fresh transfer rate (%)	309/1619 (19.09)	237/1390^3 ^(17.05)		NS
Subjects with fresh transfer	309	237		-
Subjects fresh transfer rate (%)	309/341 (90.62)	237/249 (95.18)		<0.05
Embryos cryopreserved	640	560^4^		-
Embryo cryopreservation rate (%)	640/1619 (39.53)	560^4^/1465^1 ^(38.22)		NS
Subjects with cryopreservation	191	166^5^		-
Subjects cryopreservation rate (%)	191/309 (61.81)	166/237 (70.04)		0.05
		Day 3	Day 5	
*Proportion IVF/ICSI as in vitro *fertilization procedure (%)				
IVF		12/135 (8.89)	10/102 (9.80)	NS
ICSI		123/135 (91.11)	92/102 (90.20)	NS
Mean No. cumulus-oocyte complexes (SD)		9.18 (5.92)	14.52 (6.25)	<0.05
Mean No. metaphase II oocytes (SD)		6.00 (4.31)	11.01 (6.08)	<0.05
Mean No. two pronuclei oocytes (SD)		4.73 (3.00)	10.05 (4.06)	<0.05
Embryos on day 3		615	1000	-
Embryos on day 5		NA	775	-
Embryos transferred fresh		135	102	-
Embryo fresh transfer rate (%)		135/615 (21.95)	102/775 (13.16)	<0.001
Embryos cryopreserved		154	363	
Embryo cryopreservation rate (%)		154/615 (25.04)	363/775 (46.84)	<0.001
Subjects with cryopreservation		80	84	-
Subjects cryopreservation rate (%)		80/135 (59.26)	84/102 (82.35)	<0.001

^1 ^including 75 day 3 embryos from 6 subjects without fresh transfer.

^2 ^including 41 day 5 embryos from 2 subjects without fresh transfer.

^3 ^available day 3 embryos for subjects with fresh day 3 transfer and available day 5 embryos for subjects with fresh day 5 transfer.

^4 ^including 43 embryo cryopreserved on day 3 and 5 in subjects without fresh transfer.

^5 ^including 2 subjects with cryopreserved embryos without fresh transfer.

Overall, no differences were observed in the total number of cumulus-oocyte complexes, MII oocytes or 2PN oocytes. However, a significantly lower number of cumulus-oocyte complexes, MII oocytes and 2PN oocytes were available in the subgroup of patients with a transfer on day 3. Patients with the fixed day 5 approach had a lower subjects' fresh transfer rate (90.62% vs. 95.18%, p < 0.05) but the embryo fresh transfer rate and the embryo cryopreservation rate was similar. For patients in the day 3 or day 5 group, a higher embryo fresh transfer rate and a lower embryo cryopreservation rate were observed in patients with a transfer on day 3. The data reported separately for the day 3 and day 5 subgroups only included cycles with embryo transfer as cancelled cycles (n = 12) cannot be allocated to either one of the two subgroups.

### Fresh embryo transfer: outcome (Table 3)

**Table 3:** Fresh embryo transfer outcome

	eSET with elective day 5 transfer		eSET with transfer day 3 or 5		Statistical significance
	/cycle	/ET	/cycle	/ET	/cycle
Pregnancy rate (positive hCG)	141/341 (41.35)	141/309 (45.63)	95/249 (38.15)	95/237 (40.08)	0.45
Clinical pregnancy rate	121/341 (35.48)	121/309 (39.16)	79/249 (31.73)	79/237 (33.30)	0.38
Ongoing pregnancy rate	118/341 (34.60)	118/309 (38.19)	73/249 (29.32)	73/237 (30.80)	0.18
Pregnancy loss					
Miscarriage	14	14	10	10	-
Ectopic pregnancy	5	5	1	1	-
Live birth rate	112/341 (32.84)	112/309 (36.25)	72/249 (28.92)	72/237 (30.38)	0.32
Mulitple pregnancy rate	4/341 (1.17)	4/309 (1.29)	0/249 (0)	0/237 (0)	0.16
			Day 3/cycle (%)	Day 5/cycle (%)	
Pregnancy rate (positive hCG)			47/135 (34.81)	48/102 (47.06)	0.08
Clinical pregnancy rate			39/135 (28.89)	40/102 (39.26)	0.10
Ongoing pregnancy rate			36/135 26.67)	37/102 (36.27)	0.16
Pregnancy loss					
Miscarriage			6	4	-
Ectopic pregnancy			1	0	-
Live birth rate			36/135 (26.67)	36/102 (35.29)	0.16
Mulitple pregnancy rate			0/135 (0)	0/102 (0)	1

No differences were observed in terms of clinical pregnancy rate, ongoing pregnancy rate or live birth rate between the fixed day 5 and the day 3 or day 5 transfer policies. These outcome parameters also did not differ between the two subgroups within the day 3 or day 5 transfer policy. Multiple pregnancies were only observed in the fixed day 5 approach (1.29%) but did not statistically higher than in the other transfer approach group.

### Frozen embryo transfer: embryology data (Table 4)

**Table 4:** Efficiency of cryopreservation and pregnancy potential of surviving embryos

	eSET with elective day 5 transfer	eSET with transfer day 3 or 5		Statistical significance
No. of embryos cryopreserved	640	517		
No. of thawing cycles	188	143		
No. of embryos thawed	286	229		
No. of embryos surviving	200	150		
Transferrable survival rate	200/286 (69.9)	150/229 (65.5)		NS
Subjects with thawed transfers	109	71		
Total number of thawed transfers	171	125		
Total number of clinical pregnancies	26	21		
Transfer specific pregnancy rate^1^	26/171 (15.2)	21/125 (16.8)		NS
Subject specific pregnancy rate^2^	26/109 (23.9)	21/71 (29.6)		NS
		Day 3	Day 5	
No. of embryos cryopreserved		154	363	
No. of thawing cycles		41	102	
No. of embryos thawed		84	145	
No. of embryos surviving		39	111	
Transferrable survival rate		39/84 (46.4)	111/145 (76.5)	<0.001
Subjects with thawed transfers		26	45	
Total number of thawed transfers		28	97	
Total number of clinical pregnancies		4	17	
Transfer specific pregnancy rate^1^		4/28 (14.3)	17/97 (17.5)	NS
Subject specific pregnancy rate^2^		4/26 (15.4)	17/45 (37.8)	0.05

^1 ^Total number of pregnancies/total number of thawed transfers

^2 ^Total number of pregnancies/number of subjects with thawed transfers

An overall embryo transfer rate of 69.9% was observed in the fixed blastocyst group which was not significantly different from the overall embryo transfer rate of 65.5% was observed for day 3 and day 5 embryos combined in the other group. The embryo transfer rate was statistically higher in the day 5 subgroup as compared to the day 3 subgroup (76.5% vs. 46.4%, p < 0.001). No differences were observed in the transfer specific or subject specific clinical pregnancy rates.

### Frozen embryo transfer: outcome (Table 5)

**Table 5:** Frozen embryo transfer outcome

	eSET with elective day 5 transfer		eSET with transfer day 3 or 5				Statistical significance
	/cycle (n = 188)	/ET (n = 171)	/cycle (n = 143)		/ET (n = 123)		
Positive hCG (%)	34/188 (18.06)	34/171 (19.88)	30/143 (21.00)		30/123 (24.39)		NS
Clinical pregnancy rate (%)	26/188 (13.83)	26/171 (15.20)	21/143 (14.69)		21/123 (17.07)		NS
Ongoing pregnancy rate (%)	24/188 (12.77)	24/171 (14.04)	18/143 (12.56)		18/123 (14.63)		NS
Pregnancy loss							
Miscarriage	2/26 (7.69)		3/20 (20)				NS
Ectopic pregnancy	0		1/30 (3.33)				NS
Single embryo transfer (%)	142/171 (83.0)		96/123 (78.05)				NS
Multiple pregnancy rate (%)	2/24 (8.33)		1/18 (5.56)				NS
			Day 3		Day 5		
			/cycle (n = 41)	/ET (n = 26)	/cycle (n = 102)	/ET (n = 97)	
Positive hCG (%)			5/41 (12.20)	5/26 (19.23)	25/102 (24.51)	25/97 (25.77)	NS
Clinical pregnancy rate (%)			4/41 (9.76)	4/26 (15.38)	17/102 (16.67)	17/97 (17.53)	NS
Ongoing pregnancy rate (%)			4/41 (9.76)	4/26 (15.38)	14/102 (13.72)	14/97 (14.43)	NS
Pregnancy loss							
Miscarriage			0		3/17 (17.6)		NS
Ectopic pregnancy			0		1/25 (4)		NS
Single embryo transfer (%)			13/26 (50.0)		83/97 (85.6)		<0.001
Multiple pregnancy rate (%)			¼ (25)		0		NS

Single embryo transfer was performed in 83% of all frozen blastocyst transfers in the fixed day 5 group. Single blastocyst transfers in the day 5 subgroup of the day 3 or day 5 group counted for 85.6% of the cycles which was significantly more than the 50% SET observed in the day 3 subgroup. The ongoing pregnancy rate per frozen embryo transfer in the fixed day 5 groups was 14.04%, which was not different from the overall ongoing pregnancy rate of the day 3 or day 5 group (14.63%). Multiple pregnancy rates were not different between the different approaches.

### Summary and projected outcome (Table 6)

**Table 6:** Summary and projected pregnancy rate compensating for the double counting of pregnancies in case a subject has more than one pregnancy

	eSET with elective day 5 transfer	eSET with transfer day 3 or 5		Statistical significance
No. of fresh cycles	341	249		
No. of thawed cycles	188	143		
Subjects with fresh or thawed clinical pregnancy	147	100		
Subjects with fresh or thawed ongoing pregnancy	142	91		
Number of embryos unthawed^1^	38	98		
Clinical pregnancy rate thawed embryos	26/286 (9.09)	21/229 (9.17)		NS
Ongoing pregnancy rate thawed embryos	24/286 (8.39)	18/229 (7.86)		NS
Projected number subjects with clinical pregnancy^2^	3.45	9.68 (1.24+8.44)		
Projected number subjects with a ongoing pregnancy^2^	3.19	8.20 (1.24+6.96)		
Projected cumulative clinical pregnancy rate^3^	150.45/341 (44.12)	109.68/249 (44.04)		NS
Projected cumulative ongoing pregnancy rate^3^	145.19/341 (42.58)	99.20/249 (39.84)		NS
		Day 3	Day 5	
No. of fresh cycles		135	102	
No. of thawed cycles		41	102	
Subjects with fresh or thawed clinical pregnancy		43	57	
Subjects with fresh or thawed ongoing pregnancy		40	51	
Number of embryos unthawed^2^		26	72	
Clinical pregnancy rate thawed embryos		4/84 (4.76)	17/145 (11.72)	NS
Ongoing pregnancy rate thawed embryos		4/84 (4.76)	14/145 (9.66)	NS
Projected number subjects with clinical pregnancy		1.24	8.44	
Projected number subjects with a ongoing pregnancy		1.24	6.96	

^1 ^Number of embryos unthawed for subjects with no fresh or thawed pregnancy

^2 ^Projected from the unthawed material among subjects without a fresh or thawed pregnancy, assuming that the pregnancy rate will be the same as for the already thawed material

^3 ^Fresh pregnancies, thawed pregnancies and projected number of pregnancies/number of subjects

The clinical and ongoing pregnancy rate per thawed embryo was not different between the two groups. Neither was the projected cumulative clinical and ongoing pregnancy rate different between the elective day 5 group (44.12%; 42.58%) versus the day 3 or day 5 group (44.04%; 39.84%).

## Discussion

This study has shown that a single blastocyst transfer policy can be decided before the initiation of the in vitro fertilization (IVF) cycle. A day 3 or day 5 embryo transfer policy based on day 3 embryo evaluation does not increase cycle outcome. It merely divides the patient population based on their ovarian response and reduces the risks of a cancelled embryo transfer.

Randomized controlled trials have shown that a blastocyst transfer policy results in a significantly higher live birth rate [5,11] However, the effect of a fresh blastocyst transfer policy on the cumulative pregnancy rate, including cryo-cycles has not been evaluated in these trials.

A recent prospective trial by Guerif et al., [12] did include embryo cryopreservation by slow freezing technique when comparing a single day 2 with a single blastocyst transfer policy. Although blastocyst transfer yielded a higher live birth rate in the fresh cycles, it appeared to result in the same cumulative pregnancy rate after adding cryo-cycles. The authors concluded that improvements in blastocyst cryopreservation were needed in order to claim superiority of the blastocyst transfer policy. The implementation of the vitrification technique may provide such effect, however it would also further increase cleavage stage cryopreservation outcome.

A meta analysis concludes that vitrification appears to be associated with a significantly higher postthawing survival rate than slow freezing [13]. The superiority of vitrification is also illustrated by comparing vitrification outcome in this study with previously published data on blastocyst slow-freezing at the same centre [14]. A reported embryo transfer rate ranging between 48.9% and 52.2% after slow freezing [14] has been substantially increased to rates varying between 69.9% and 76.5% after vitrification.

The question whether cumulative pregnancy chances are affected by a cleavage stage embryo transfer versus blastocyst culture and blastocyst transfer based on a day 3 embryo quality evaluation remained unanswered. This study showed that such a embryology based policy mainly divides the patient population based on their ovarian response. The inherent reduced reproductive potential is illustrated by the low transferrable embryo survival rate of 46.4% compared to 65.1% as previously published in elective day 3 population in the same centre [15]. An alternative interpretation of the dataset is that a day 5 transfer is not required for a large proportion of the patients as the day 3/day 5 subgroups comparison for pregnancy rates are comparable.

In this study we used an aseptic method for the vitrification of embryos. Although the transmission of infectious diseases through cryopreservation and storage of embryos has never been reported, such possibility exists under experimental conditions [7,16]. Therefore, the application of hermetically sealed containers or secondary enclosure (for cryovials and open vitrification systems) is suggested [16]. The hermetically sealed device used in this study has the advantage that both vitrification and storage are perfomed under 'closed' conditions. Aseptical vitrification using open devices would require sterilization of liquid nitrogen and a secondary enclosure for storage [17].

For the vast majority of patients, the single embryo transfer policy was based on the Belgian law imposing a SET policy in women less than 36 years [18]. No strict SET policy was applied in the thawed embryo transfer cycles, with significantly more DET (double embryo transfer) in the frozen day 3 embryo transfers. The higher proportion of DET in the frozen day 3 cycles (50%) as compared to the frozen blastocyst cycles (14.6%) can be explained by a lower expected implantation rate and resulted in a similar ongoing pregnancy rate per transfer (15.38% vs. 14.43%). The p value for the subject specific clinical pregnancy rate comparing the day 3 and day 5 embryo transfer reaches 0.05, although no difference has been observed in the pregnancy rate per transfer. The high subject specific pregnancy rate in the day 5 group appears to be due to the significantly higher transferrable survival rate and the embryo transfers performed.

Although no differences were observed in patients' and treatment characteristics, the retrospective design remains a weakness. The allocation of patients to one or the other group is based on the view of the individual physician in the centre and the preferences of the patient. Establishing a prospective randomized controlled trial will generate anxiety amongst patients and clinicians. This retrospective data will be reassuring for centers wishing to investigate this subject further.

## Competing interests

The authors declare that they have no competing interests.

## Authors' contributions

DS conceived of the study, performed the statistical analysis and wrote the manuscript. LVL coordinated the laboratory aspects of embryo cryopreservation and participated in the outcome evaluation. EVDA coordinated the laboratory aspects of embryo cryopreservation. MC helped to draft the manuscript. GV helped to draft the manuscript. PD conceived of the study and participated in its design and coordination and helped to draft the manuscript. All authors read and approved the final manuscript.
